# Reconsidering “Unprotected” and HIV Risk in the Twenty-First Century

**DOI:** 10.3389/fimmu.2016.00209

**Published:** 2016-06-08

**Authors:** Brian C. Kelly

**Affiliations:** ^1^Sociology, Purdue University, West Lafayette, IN, USA

**Keywords:** risk assessment, HIV, MSM/Ms, condoms, unprotected sex

Conceptualizations of HIV risk have changed over the 35 years since the identification of a cluster of mortality among gay men in Los Angeles (1). From these early days, condoms have served as a critical primary prevention tool. The entrenchment of condom use as the central prevention strategy during the ensuing years left a legacy by which it remained the focal point in considerations of risk. Yet, increasingly during the intervening years, concerns arose about the absence of condoms as a marker of risk. Men increasingly questioned a “one size fits all” approach to defining risk and the impact that had on their lives. Jin and colleagues (2) have provided us with useful empirical evidence for conversations about the nature of risk in the sexual encounters of men who have sex with other men, and such approaches are more feasible with methodological advances facilitating refined data collection. The pattern of sexual partnering described in their results explains why they assert “any condomless anal intercourse is no longer an accurate measure of HIV sexual risk behavior.” Simply put, risk reduction efforts are widespread during episodes of condomless anal sex among these men.

In response to the desire to provide alternative strategies to condom use, numerous harm reduction practices emerged over time. Serosorting, negotiated safety, strategic positioning, and withdrawal before ejaculation all became incorporated into the sexual routines of gay men and other MSM even prior to the more recent biomedical approaches, such as PrEP and viral load sorting. Importantly, many of these risk reduction methods emerged in a bottom-up fashion and serve as a reminder of the critical role that communities themselves play in public health promotion. The evidence provided by Jin and colleagues affords an understanding of how HIV-negative men approach sexual encounters with respect to the range of risk reduction options available to them. Among the HIV-negative men they followed over the course of several years, six out of seven episodes of condomless anal intercourse occurred with a regular HIV-negative partner. This critically underscores that most condomless anal sex occurs within the context of a regular sexual relationship with someone who is known to be HIV-negative, which alone is an important indicator that risk is misspecified when “unprotected anal intercourse” is defined simply by a lack of condom use. But, the authors further provide us with information about the use of other risk reduction strategies during condomless sex, both with casual partners and with regular partners of unknown or discordant serostatus. They find that HIV-negative men who have HIV-positive regular partners are heavily reliant upon evaluating viral load and condomless anal sex most often occurred when their partner had an undetectable viral load. This consideration of viral load is a critical component of the wider treatment as prevention strategy indicating that an HIV-positive partner’s undetectable viral load considerably reduces risk of transmission (3, 4); these men strategically put this information to use with serodiscordant regular partners. In addition, the results indicate that HIV-negative men rely heavily upon strategic positioning and withdrawal as risk reduction techniques during condomless sexual encounters with status unknown or serodiscordant partners. While acknowledging that these strategies are by no means foolproof, they do confer an element of protection beyond instances in which such strategies are not used (5), further complicating notions of protection and its absence.

A more holistic consideration of risk is even more important as biomedical advances have more fully diminished the need to solely focus on condoms in prevention among gay men and other MSM. This approach has been institutionalized with the U.S. CDC’s 2014 move to not equate condomless sex with unprotected sex. Yet, the paper by Jin and colleagues is also implicitly an important reminder of the crucial roles that testing and treatment continue to play in inhibiting the transmission of HIV with these risk reduction strategies. While the usage of the sexual risk reduction strategies have rendered notions of condomless sex as “unprotected” inaccurate, these strategies are undoubtedly optimized when current, correct information about serostatus and viral load are available. In the absence of up-to-date information, the decision-making processes underlying the intent of these strategies are undermined. For these reasons, the advocacy of strategies that provide alternatives to condom use must be accompanied by the advocacy of *regular* testing and treatment. Increasing HIV testing among MSM recently has been highlighted as critical for achieving substantial reductions in HIV incidence (6), and the promotion of testing as often as quarterly is a cost-effective public health strategy (7). Yet, rates of testing among MSM remain suboptimal even in well-resourced regions (8), and disparities in resources for testing and treatment remain considerable; not all men have similar opportunities to avail themselves of such resources. Nonetheless, the widespread use of risk reduction strategies highlights the foundational role of the accuracy of information provided by regular testing and treatment in the navigation of risk and the need to promote such engagement with health-care professionals.

Reconsiderations of risk are critical during the course of any epidemic as a scientific knowledge base is developed. Yet, reconsiderations of condomless sex as unprotected are not simply a matter of epidemiology or the production of medical and scientific knowledge. The prospect of more nuanced considerations of risk has ramifications in the personal lives and experiences of the men who consider alternatives to condom use. Changes in the way health professionals think about protection and risk provide for many men a validation in the expansion of partnering options, the enhancement of intimacy within couples, and the provision of a greater sense of agency over sex lives. Thus, beyond the pragmatic concerns of disease prevention among health professionals, prevention strategies born out of alternative notions of risk have deeply meaningful implications for how romantic and sexual partnering is experienced by gay men. More nuanced assessments of risk will not only simply lead to better science but also lend legitimacy to the experiences gay men and other MSM are striving for, which will have a broader impact on their well-being.

## Author Contributions

The author confirms being the sole contributor of this work and approved it for publication.

## Conflict of Interest Statement

The author declares that the research was conducted in the absence of any commercial or financial relationships that could be construed as a potential conflict of interest.
